# A Critical Look at the Crystal Structures of cAMP-Dependent Protein Kinases

**DOI:** 10.3390/kinasesphosphatases3030019

**Published:** 2025-09-11

**Authors:** Alexander Wlodawer, Pawel Rubach, Zbigniew Dauter, Wojciech Dec, Wladek Minor, Dariusz Brzezinski, Mariusz Jaskolski

**Affiliations:** 1 Laboratory of Cell Biology, Center for Cancer Research, National Cancer Institute, Bethesda, MD 20892, USA; 2 Department of Molecular Physiology and Biological Physics, University of Virginia, Charlottesville, VA 22908, USA; 3 Institute of Information Systems and Digital Economy, Warsaw School of Economics, 02-554 Warsaw, Poland; 4 HKLResearch, Charlottesville, VA 22908, USA; 5 Department of Computational Biophysics and Bioinformatics, Jagiellonian University, 31007 Krakow, Poland; 6 Doctoral School of Exact and Natural Sciences, Jagiellonian University, 30-387 Krakow, Poland; 7 Institute of Computing Science, Poznan University of Technology, 60-965 Poznan, Poland; 8 Institute of Bioorganic Chemistry, Polish Academy of Sciences, 61-704 Poznan, Poland; 9 Department of Crystallography, Faculty of Chemistry, Adam Mickiewicz University, 61-614 Poznan, Poland

**Keywords:** kinases, structure quality, Protein Data Bank (PDB), PDB-REDO, KLIFS database

## Abstract

We have evaluated the quality of all 325 deposits in the PDB (as of December 2024) that correspond to (or contain) the catalytic domain of cAMP-dependent protein kinases (PKA). Detailed analysis was possible for 289 deposits of crystal structures that included not only the atomic coordinates but also structure factors. These structures represent 35 years of studies, and it is not surprising that the more recent structures are generally of better quality than the older ones. We did not encounter deposits with very severe problems, although some minor problems were found. To assess whether a uniform method of structure re-refinement, as implemented in the pipeline and website PDB-REDO, leads to significant improvement of structural models, we compared structure quality indicators for the originally refined structures and their counterparts resulting from PDB-REDO refinement. The re-refinement procedure significantly improved only some older structures, while its success was generally limited. We paid particular attention to the quality of small-molecule ligands, finding that most of them fit the electron density very well. This type of analysis helps identify the highest quality structures among many deposits for certain protein families and, thus, could be extended to other groups of proteins as well.

## 1. Introduction

Maintaining the highest standard of the deposits in the Protein Data Bank (PDB) [1–3] is critical for various reasons. For instance, biologists often use individual structures deposited in the PDB to modify protein function. The whole contents of the PDB have been used for training artificial intelligence-based structure prediction systems such as AlphaFold [4], RoseTTAFold [5], ESMFold [6], and other similar programs. Another project dependent on the utilization of a large number of structures of enzymes belonging to a particular superfamily led recently to a very comprehensive description of the enzymatic mechanism of serine proteases, based on a titanic analysis of over one thousand PDB coordinate sets of apoenzymes and their complexes with a variety of ligands [7]. However, that work did not address directly the relative quality within such multiple sets of deposits.

Our attention was focused on kinases, which are enzymes of crucial importance for maintaining cellular functions and which are targeted by a number of very successful drugs against several diseases, including cancer and various inflammatory conditions [8–10]. Over 6700 structures of kinases are available in the PDB, as well as in a specialized database named KLIFS [11]. Having calculated the quality indicators for these structures, we selected a particular group of medicinally essential enzymes to investigate the quality of their crystal structures in greater detail. The 325 PDB deposits chosen for this purpose represent the catalytic subunits of cAMP-dependent protein kinase A (PKA) from mouse [12–14] and closely related enzymes from other mammalian species, with sequence identity of 95% or higher. Although more numerous sets of models may be found in the PDB for other proteins, for instance, hen-egg lysozyme (over 1250 deposits), bovine trypsin inhibitors (over 1600 deposits), or hemoglobin (over 8350 deposits), our choice was based on both the direct medicinal importance of the enzyme family to be studied, as well as the fact that these enzymes, unlike the other proteins mentioned above, have not been used in a significant way for testing new structure determination methodologies and refinement procedures. The medicinal importance of PKAs cannot be overemphasized, as these enzymes and the pathways they control have been used as targets for the design of anticancer drugs [15]. The crucial role of PKA in signaling in cancer [16] has been extensively studied, but interfering with its activity leads to significant side effects. Nevertheless, inhibitors of PKA are still being actively developed [17]. Additionally, PKA was the prototype for a number of other protein kinases that ultimately became very important drug targets [18]. We hope that the analysis of the PKA structures presented in this manuscript will guide researchers studying the properties of these enzymes (especially those with no background in structural biology) toward identifying the most reliable and trustworthy structural models in this group.

Another advantage of choosing this family for an initial study is that, over more than 30 years, these structures have been determined in different laboratories, using various data collection and refinement protocols. Except for the earliest structure in this family (PDB ID 2cpk; [12]), almost all other structures were solved by molecular replacement. Nevertheless, as shown below, the quality of the models varies quite significantly.

Our analysis is limited to only crystal structures of the PKA enzymes, for which quality assessment criteria are strictly and stringently defined (see below), because such models are usually used as targets for structure-guided drug design, and because so far crystallography has furnished the most significant number of structure models of PKAs (325), compared to electron microscopy (9). As part of this project, we compared the original PDB deposits with structures re-refined by the automated protocol PDB-REDO [19], to assess whether such standardized procedures could significantly improve the quality of the models. We also compared the quality of PKA structures with those of other kinases from the KLIFS database, some of which are even more significant from a biomedical perspective.

The present analysis of the PKA enzymes continues our similar efforts devoted to medicinally relevant PDB structures, such as metallo-β-lactamases [20], SARS-CoV-2 proteins [21,22], or L-asparaginases [23].

## 2. Results

### 2.1. Overall Quality of the PKA Models and the Underlying Data

**Figure 1.**
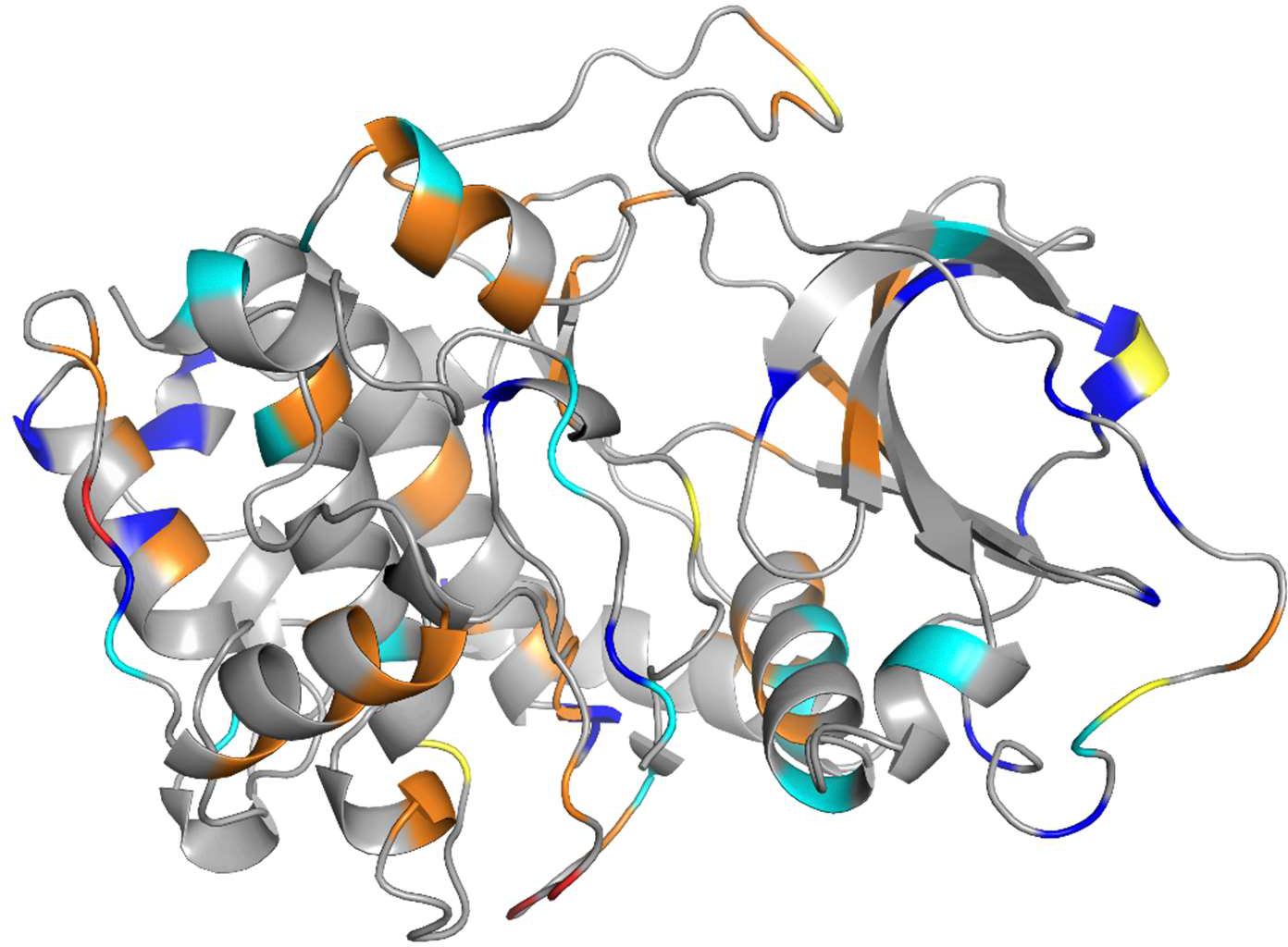
Cartoon diagram of the main chain of the catalytic domain of mouse PKA (2cpk; [12]), the first structure determined for this protein almost 35 years ago. The protein main chain is shown in gray, with residues colored according to the type of outlier identified in the 2cpk deposition: Ramachandran outliers in yellow, bond angle outliers in cyan, side-chain outliers in blue, and steric clashes in orange. The figure provides an example of visualization of potential structural issues and was generated using a newly developed PyMOL plug-in.

We found 334 deposits in the PDB that contained the catalytic domains of PKA. The structure determined first is shown in Figure 1. Although this legacy structure does not meet the quality standards of more recently determined PKA crystal structures, the original publication describing it [12] has been cited over 1600 times, testifying to its importance.

Among them were 325 crystal structures that contained one or more copies of the catalytic domain, in some cases as part of a full-length enzyme that also included the regulatory domain. For each structure, we calculated the global parameter PQ1 as a single-number quality indicator expressed in terms of the ranking against the whole PDB [24].

The structures of PKA and its modifications analyzed here span a wide range of resolution of the diffraction data, from atomic (1.03 Å) to very low resolution (4.75 Å), with about 2/3 of the structures determined at the resolution of 2 Å or better. The *R*_merge_ (or sometimes *R*_scale_) measure of the diffraction data quality was reported for only 184 datasets and ranged from 0.026 to 0.22, with an additional outlier 5j5x, reporting a value of 0.366. Most structures were generally well refined, with the values of *R*_free_ (as reported by the authors and very strongly correlated with diffraction data resolution) ranging from 0.141 to 0.371.

The number of PDB entries of the target enzymes that did not include diffraction data was 33 (Table 1), all deposited before 2008, and the PQ1 values for these models were estimated based only on the structure geometry data. Additionally, 8 datasets that included structure factor data did not identify reflections used for *R*_free_ calculation, thus such calculations (performed by PDB-REDO [19]) had to select a new set of test reflections that might not be an independent (“free”) and representative of the set used by the original authors. For those structures, only *R*_free_ (if any) claimed by the authors was reported by the PDB, whereas the DCC assessment used for structure factor validation did not report its value.

**Figure 2.**
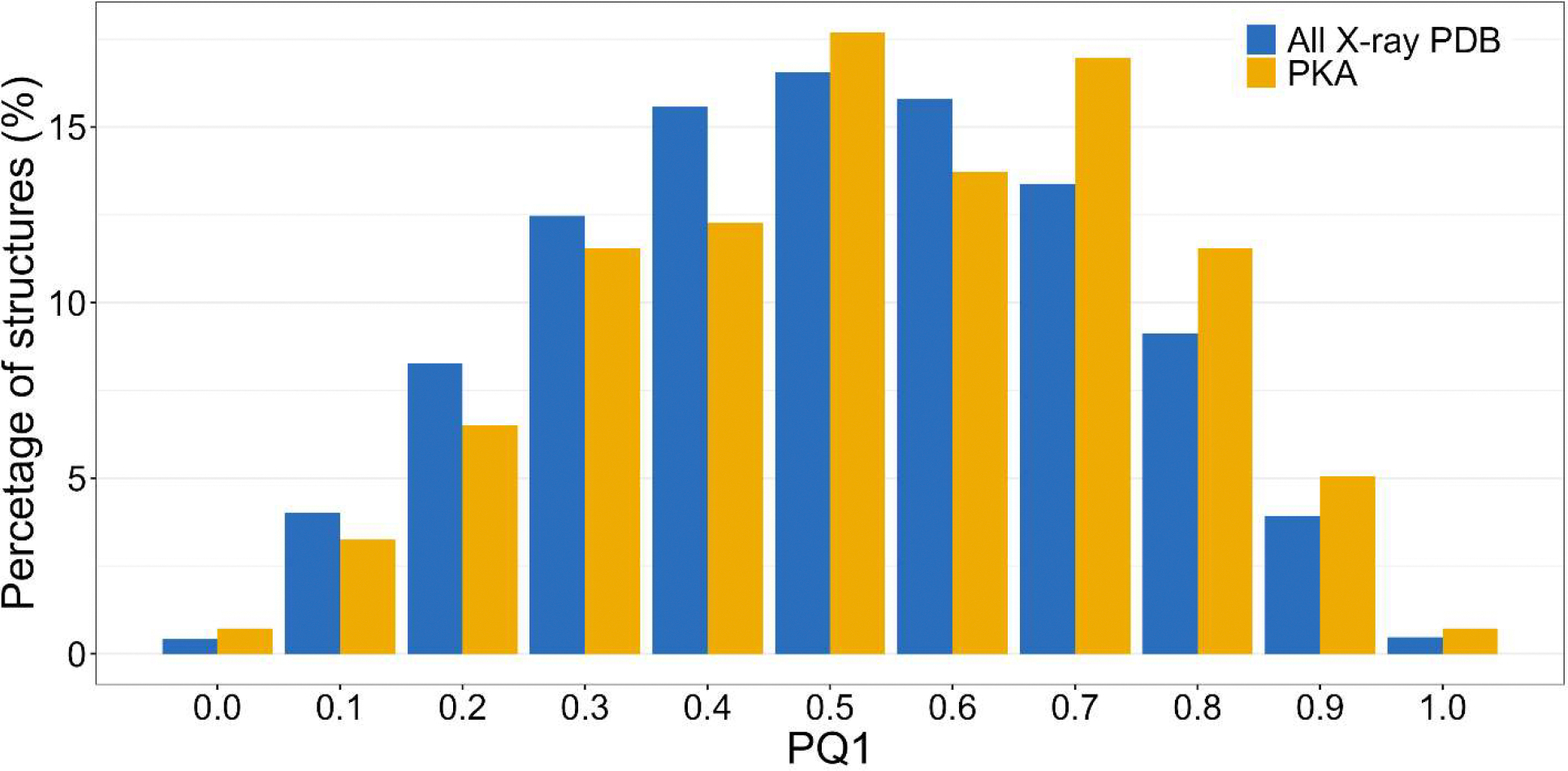
A histogram showing the fractional distribution of the PQ1 parameter in all crystal structures in the PDB (blue) and in the structures of PKA (yellow).

Not surprisingly, considering that the structures of PKA were determined by molecular replacement from well-established models, their quality compared to the whole contents of the PDB was better than average (Figure 2). Only 125 structures are characterized by PQ1 less than 0.5, whereas almost 200 structures fall into the “better than the average” category (Supplementary spreadsheet File S1). Many lower-quality structures were determined before 2008 and deposited without diffraction data. For that reason, the values of PQ1 for those structures could only be estimated very roughly, and their significance is less clear. Also, it is impossible to directly evaluate the fit of the atomic coordinates of those models to the respective electron density maps. We decided to keep these datasetss in this analysis, although we are fully aware that they cannot be fully validated in the same way as the more recent structures.

### 2.2. Identification of Problems and Their Treatment by PDB-REDO

All models with available structure factors (289 out of 325) were re-refined by PDB-REDO and the results are available at https://pdb-redo.eu/ (accessed on 1 April 2025). In some cases, the re-refinement led to significantly improved fit to the electron density (as judged by lower values of RSRZ and *R*_free_) and to improved clashscore values (Table 2; Supplementary spreadsheet File S1). Since no major problems with the Ramachandran angles were present in these models, the improvement of that aspect was usually insignificant. The re-refinement also led to the occasional removal of spurious water molecules but not to the addition of unmodeled solvent molecules.

Although the vast majority of PKA structures do not exhibit significant problems, some deposits have evident shortcomings and flaws of different severity. An example is the structure of the (73–244)Ria:C holoenzyme of PKA (PDB ID 3pvb; [25]). The coordinates and *B*-factors were refined at 3.3 Å resolution to depositor-provided *R*_free_ of 0.290, although the DCC-reported value is 0.340. The structure factors deposited in the PDB extend to 2.5 Å resolution, although their completeness at the highest resolution is low. Rerefinement with PDB-REDO (at 3.3 Å) yielded *R*_free_ of 0.326, with equally poor geometry and fit to the electron density. The reason for these discrepancies is not obvious.

Refinement of the structure of the C-terminal domain of PKA at 2.2 Å resolution (PDB ID 3e8c; [26]) converged to *R*_free_ of 0.290 (depositor) or 0.286 (DCC), with other quality parameters in the average range. However, many water molecules had abnormally low *B*-factors. Refinement with PDB-REDO led to a very substantial improvement of *R*_free_ (to 0.240), a better fit of the side chains to the electron density, and removal of many unjustified water molecules.

A large discrepancy in the values of *R*_free_ [0.288 (depositor) or 0.220 (DCC)] for the 2.0 Å resolution PKA complex with phenol (PDB ID 3nx8; [27]) may be due to misidentification of the free reflection set in the deposited structure factor files. However, lowering of *R*_free_ to 0.210 by PDB-REDO with substantially improved fit of many residues to the electron density might indicate a more fundamental problem with the original refinement.

An example of a very large improvement in the refinement parameters after PDB-REDO is provided by the structure of the triple mutant of PKAB3 (PDB ID 3l9m; [28]). The values of both *R*_free_ and *R* reported by the depositors (0.270 and 0.223, respectively) were significantly higher than their DCC equivalents (0.240 and 0.210, respectively). They were further reduced (to 0.205 and 0.165, respectively) by PDB-REDO, with significant improvement of the geometric parameters and fit to the electron density. Other structures from the same series, 3l9l and 3l9n, exhibited a similar behavior.

It might be of considerable interest to investigate whether the uniform re-refinement procedures employed by PDB-REDO can improve the structure quality in a majority of cases, thus making that derivative repository an even better source for protein structure models than the PDB itself. We observed that, in some cases, structures with low PQ1 values were significantly improved by PDB-REDO, with some examples listed in Table 1. However, as expected, for structures that were initially in the top-quality range, there was only marginal improvement or, in some cases, even deterioration of the quality metric parameters. An illustration is provided by the unpublished 1.58 Å resolution structure 5n3p, in which refinement with PDB-REDO increased *R* from 0.158 to 0.179 and *R*_free_ from 0.181 to 0.205, with no residues fitting the electron density better, and eight actually fitting worse than before. Other structures exhibiting similar behavior are also listed in Table 1.

To investigate whether the poor quality observed in some kinase structures is due to the lack of proper care by depositors, we compared the structural quality of entries from the KLIFS database (excluding PKA structures) with our set of PKA structures. The comparison focused on just one metric: the DCC *R*_free_ and the *R*_free_ values recalculated after automated re-refinement using PDB-REDO. We restricted our analysis to structures deposited within the last 15 years, i.e., after January 2010.

**Figure 3.**
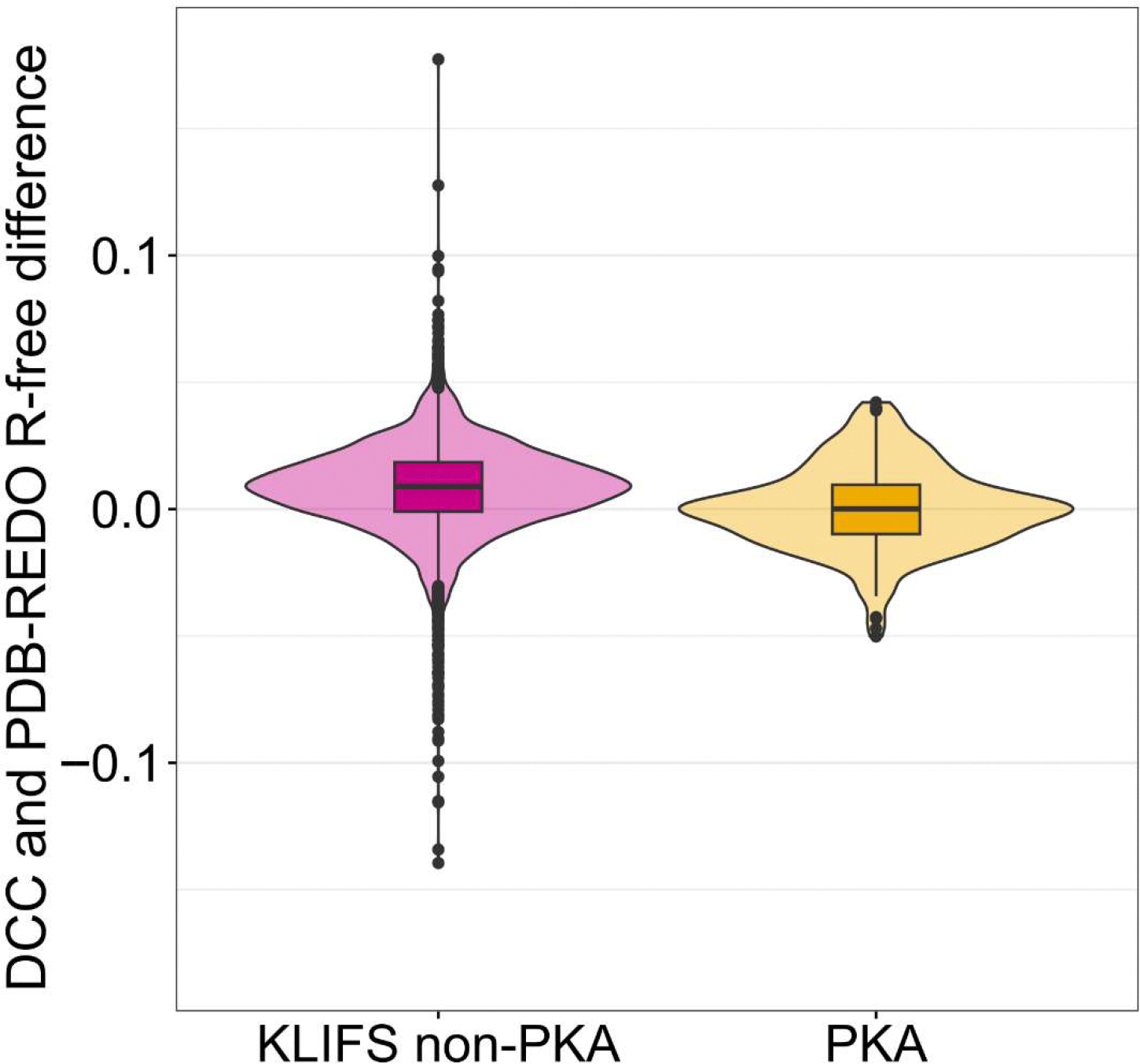
Difference between DCC *R*_free_ and PDB-REDO *R*_free_ for the KLIFS dataset without PKA (pink) and for our PKA dataset (yellow). The violin plots show the shapes (kernel density estimates) of the distributions of the respective datasets. The boxplots span *R*_free_ difference from the first to the third quartile, with the line within each box representing the median (0.009 for KLIFS without PKA and 0.000 for PKA). The lower and upper whiskers mark the distance of 1.5 × interquartile ranges (IQR) from the first and third quartile, respectively. The dots represent outliers.

DCC *R*_free_ was selected as the primary benchmark to reduce the influence of typographical errors and inconsistencies introduced by depositors or specific refinement programs. As illustrated in Figure 3, the extent of improvement in PKA structures following PDB-REDO re-refinement is lower than that observed for other kinases. Furthermore, PKA structures show virtually no quality outliers, underscoring their overall better consistency and accuracy. This observation might suggest that somewhat less attention to the quality of the final models was paid by some depositors of the larger KLIFS kinase dataset.

Interestingly, a subset of structures, exemplified by 8qcd [29], exhibited a marked decline in quality after re-refinement, not only in *R*_free_ values but also in overall structural integrity. Upon closer inspection, we discovered that this degradation stemmed from poor water modeling by PDB-REDO and the unexpected removal of all hydrogen atoms, even for a high-resolution structure determined at 1.03 Å. In this case it is not stated in the publication [29] or in PDB header if H-atom positions were refined or simply added to the final model at riding position by Phenix.refine [30].

In another case (6gzm [31], determined at 1.59 Å), almost 800 water molecules were removed (833 present in the PDB structure, 49 in the PDB-REDO structure) and the *R*_free_ value increased from 0.26 (DCC) to 0.3988 (PDB-REDO). When this result was communicated to PDB-REDO, the entry has been corrected. These findings highlight potential limitations of automated re-refinement pipelines, particularly when applied to already well-refined, high-resolution datasets.

### 2.3. Small-Molecule Ligands in PKA Structures

Since the catalytic domain of PKA contains a binding site for ATP, the majority of the analyzed structures contain this ligand or its modifications. Thus, the stereochemical quality of the ligand and its fit to the electron density contribute to the assessment of the overall quality of a particular deposit. PDB lists these properties for the “ligands of interest”, as identified by the depositors, excluding solvent and ions from such analysis. However, some ligands that bind outside of the ATP pocket are also evaluated by the PDB.

**Figure 4.**
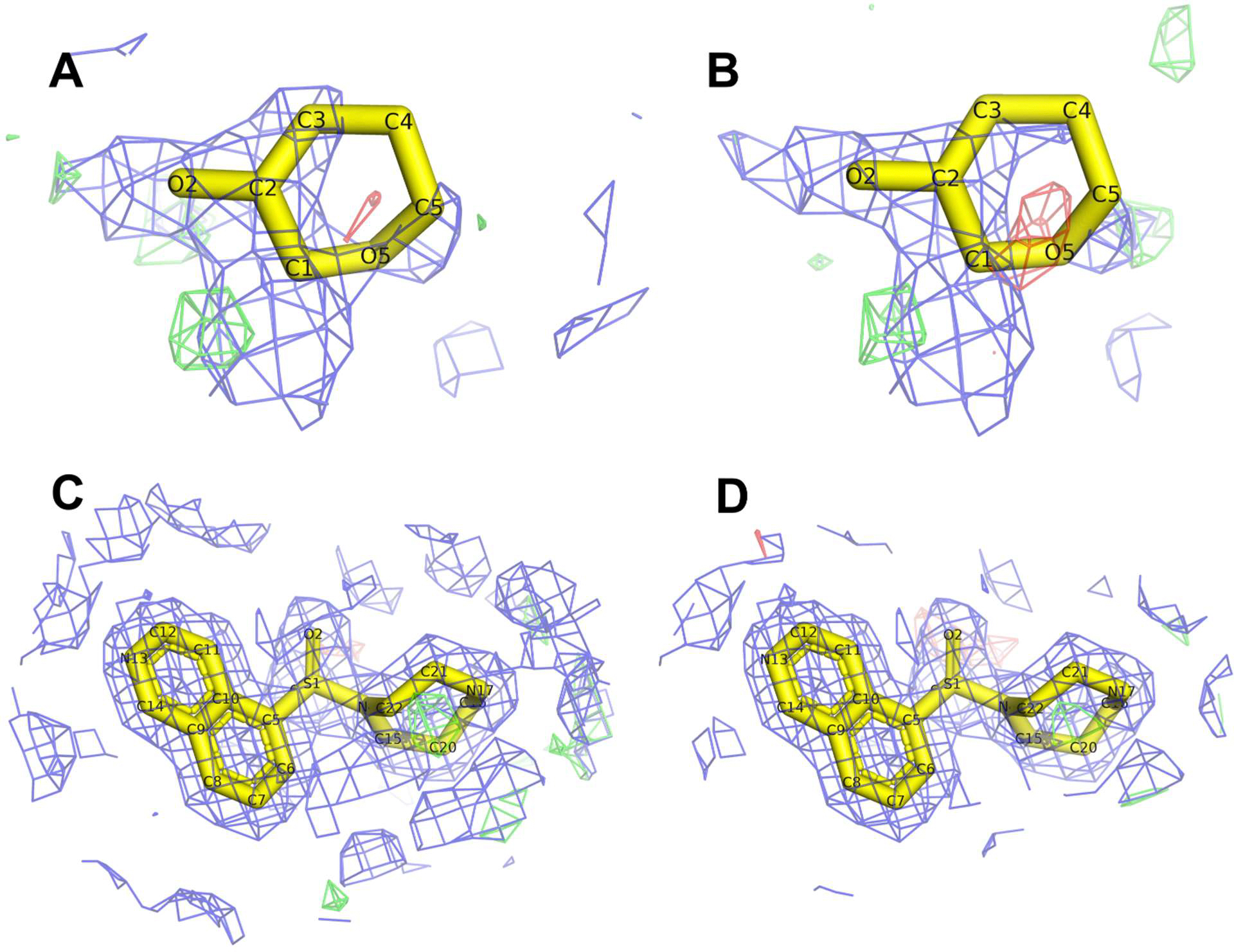
Examples of a poorly modeled ligand (5oua; [32]) (**top**) and an excellent ligand (5o0e; [32]) (**bottom**) in comparatively recent PKA structures, in the original deposits (**left**) and after re-refinement with PDB-REDO (**right**). The RIP (β-D-ribopyranose) ligand in the 5oua PDB structure (the lowest ligand quality score) (**A**) as deposited by the authors, and (**B**) after re-refinement with PDB-REDO. The M77 [5-(1,4-diazepan-1-sulfonyl) isoquinoline] ligand in the 5o0e PDB structure (the highest ligand quality score) (**C**) as deposited by the authors, and (**D**) after re-refinement with PDB-REDO. The electron density maps are carved around the ligands and contoured as follows: 2Fo-Fc (blue) 1σ, Fo-Fc (positive green, negative red) 3σ. The ligands and the electron density maps can be inspected using an interactive figure created with Molstack [33] at https://molstack.bioreproducibility.org/collection/view/Gm4amNyJ3T0hc7BEf66j/ (accessed on 1 April 2025).

Among the analyzed structures, 38 models of PKA do not contain any ligands, whereas 18 contain two or more ligands of interest. We were unable to calculate the ligand quality score for another 38 models. We accepted the PDB-calculated fit to electron density as an indicator of ligand quality. As examples, Figure 4 shows ligands with the lowest (RIP in 5oua) and the highest (M77 in 5o0e) values of ligand quality score.

The apo structure of the catalytic domain of PKA with the PDB ID 4nts [34] contains myristic acid as a ligand binding outside of the ATP pocket, but there is no clear electron density. This is fairly unusual for this class of proteins, since in almost all cases the ligands, if present, are satisfactorily modeled in the electron density maps.

## 3. Discussion

Unlike in several previous analyses of PDB crystal structures aimed at finding potential problems, such as errors in the crystallographic procedures [35], duplicate entries [36], lack of modeled solvent [37], or misassignment of critical ligands [20], to name a few, here we selected a concrete protein, well-represented in the PDB by, in general, good-quality models, to evaluate whether standardized methods of re-refinement offered by PDB-REDO could lead to further improvement of such models. Crystal structures of PKA have been accumulating in the PDB for over 30 years [12,38], and the deposits come from several laboratories. For that reason, analysis of their quality sheds some light on the gradual improvements resulting from the development of better data collection and structure refinement methods, as well as on the relative quality of PDB deposits for this selected enzyme family as compared to all other deposits of protein crystal structures.

We found that the quality of PKA deposits, as reflected by a comparison of the PQ1 parameter with the whole content of the PDB, is generally better than the average. The same can be said about the quality of the ligands of interest, the structures of which in most deposits were in excellent agreement with the experimental electron density. Nevertheless, we were able to identify a small number of structures with less significant problems and to suggest the structures of the highest quality that might be selected as representative members of this particular family. We would like to postulate that similar analyses might be worthwhile for other proteins with multiple entries in the PDB. The fact that the majority of structures determined by different hands during a comparatively long time period were found to be of high quality is very reassuring.

The PDB has curated and made publicly available over 230,000 experimentally determined protein structures, providing a rich, diverse, and high-quality dataset essential for training and validating deep-learning models, such as AlphaFold or RoseTTAFold. These experimentally determined structures enabled the elucidation of the complex relationships between amino acid sequences and their corresponding 3D structures. Without this extensive and rigorously validated structural repository, there would be no reliable ground truth to guide the development of accurate predictive algorithms.

We hope that the results presented in this manuscript could be used to make the PDB even better. Discrepancies between values calculated by the DCC and those reported by depositors could be minimized through a direct integration between refinement or pipeline software, such as Phenix v. 1.21 [39], REFMAC5 v. 5.8.0430 [40] or HKL-3000 [35,41], and the PDB deposition system. Likewise, issues such as the number of water molecules [37] could be automatically verified before deposition, improving consistency and data quality at the source.

It is essential to remember that the PDB is a repository, not a correction service—it cannot revise or improve deposited structures. While PDB-REDO provides an automated re-refinement pipeline to potentially improve structural models, this approach has inherent limitations. For instance, although PDB-REDO removes excessive or poorly supported solvent molecules, it does not add water molecules, even in cases where the original, high resolution structures lack any solvent, for which there is excellent experimental evidence. This example highlights the need for more comprehensive and context-aware refinement, ideally performed with human inspection of the results.

**Figure 5.**
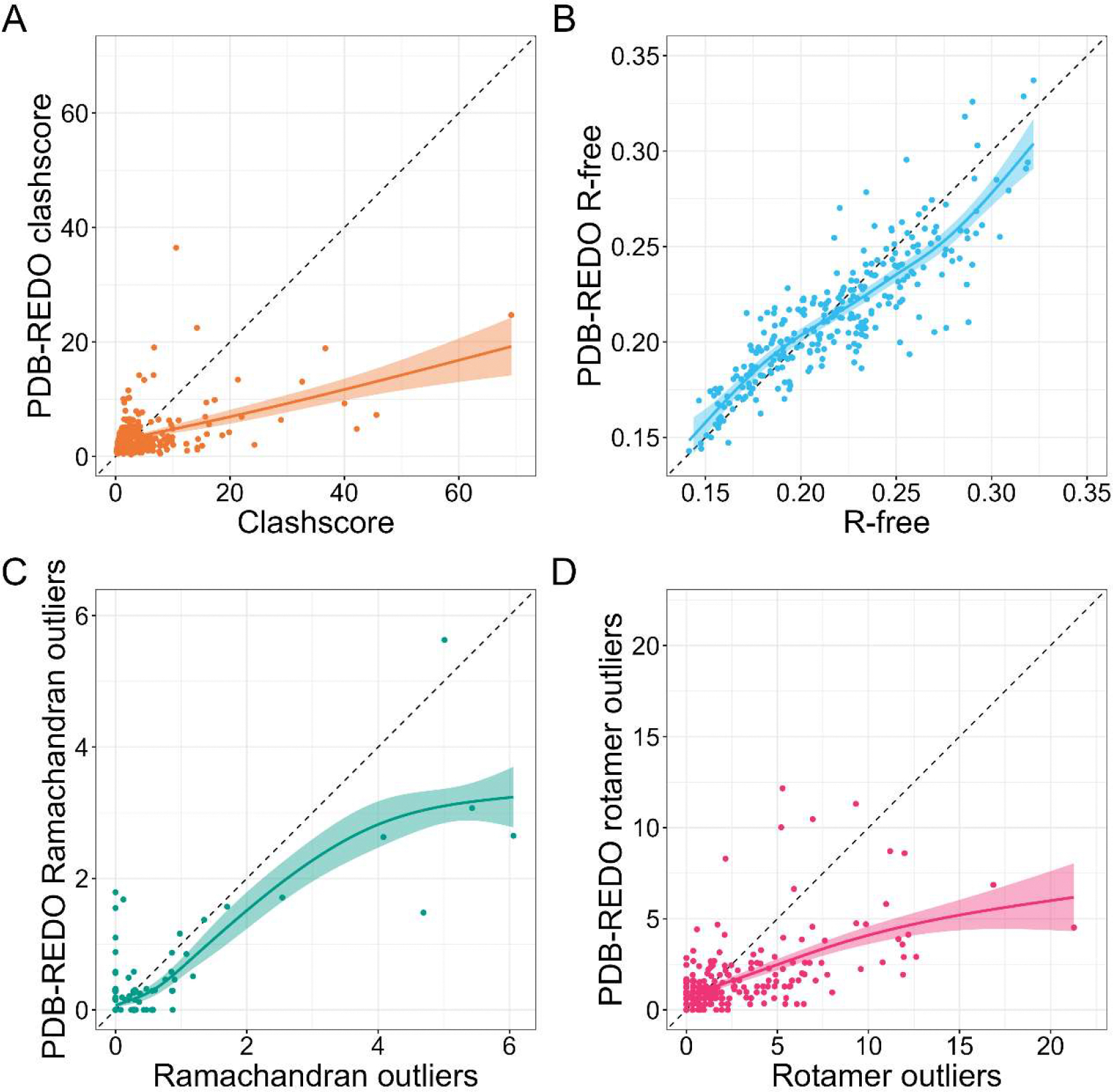
Comparison of the quality metrics for PDB and PDB-REDO models of PKA. (**A**) Clashscore, (**B**) *R*_free_, (**C**) Ramachandran outliers, and (**D**) Rotamer outliers. The trend lines on these figures were prepared using Loess regression, and the shaded area represents the confidence interval of the trend. This figure is also available in an interactive version, which allows identification of the PDB ID of each structure, at: https://bioreproducibility.org/figures/kinase/Figure 5/(accessed on 1 April 2025).

A comparison of quality metrics between PDB and PDB-REDO models, presented in Figure 5, offers an important lesson not only for structural biologists but indeed for all users of the PDB. It emphasizes the fact that improvements in *R*_free_ can sometimes come at the expense of other quality parameters. This underscores the need for a holistic evaluation of structure quality, rather than relying on a single metric.

Rather than simply highlighting errors or attributing issues to depositor oversight, our aim has been to propose practical solutions to address recurring problems observed in both PDB and PDB-REDO entries. Foremost among these is the development of a seamless interface between refinement software and the PDB deposition system. Such integration would help eliminate mistakes arising from manual data entry, typographical errors, or limited familiarity with CIF file formatting—issues that are especially common among less experienced depositors. PDB-REDO should also strive for greater compatibility with the PDB from the user’s perspective. As a minimum, PDB-REDO should provide validation reports and MTZ files that are identical in structure to those generated for the original PDB entries.

In the course of this research, we found no evidence that structures improved by PDB-REDO yield new biomedical insights. While this may be somehow disappointing, it does not preclude the possibility that such insights could emerge from other current or future experimental structures. Importantly, this work allowed us to identify various issues across multiple software tools and to propose a number of solutions aimed at improving the structure deposition process.

## 4. Materials and Methods

### 4.1. Data Collection

A search of the PDB for structures with amino acid sequence at least 95% identical to that of the catalytic domain of mouse PKA [12] identified 331 entries. The choice of the cut-off value was dictated by the need to include mutated forms of the protein and its variants from other closely related species, while excluding other kinases. The search used the clusters published by the RCSB (https://www.rcsb.org/docs/grouping-structures/sequence-based-clustering) (accessed on 1 April 2025), computed using the MMSeq2 [42] Release 15 software. Since our study was focused on crystal structures, the nine cryoEM models were excluded from this dataset, bringing its size down to 322 X-ray structures released between January 1993 (2cpk) and December 2024. Three more structures were added during the preparation of this manuscript. We also analyzed the KLIFS database [11] (available at: https://klifs.net) (accessed on 1 April 2025). We found 22 additional structures (18 crystal structures) from the PKA family, which were not included in the 95% MMSeqs2 sequence cluster computed on the RCSB site. On the other hand, the KLIFS database does not include apoenzymes and proteins from organisms other than human or mouse; therefore, our dataset contains 220 structures not listed in KLIFS.

For each structure in our dataset, the following quality parameters were extracted from the PDB: resolution, *R*_merge_, *R*_free_ [43], <I/σ> in the last shell, clashscore, rotamer outliers, Ramachandran outliers, RSRZ outliers, and number of solvent molecules. Out of the 325 crystal structures, 289 have structure factors available in the PDB and, therefore, can be re-refined, for example, by PDB-REDO [19]. The PDB-REDO *R* and *R*_free_ metrics were extracted from that resource as RFIN and RFFIN, respectively. Other PDB-REDO derived parameters that were not available directly from PDB-REDO, such as clashscore, rotamer outliers, Ramachandran outliers, and the MolProbity Score were calculated by us using MolProbity [44] from the CCP4 distribution 9.0.004 [45].

### 4.2. Structure and Ligand Quality Metrics

To systematically evaluate the retrieved PKA structures, the PDB deposits were ranked using macromolecule and ligand quality metrics (Supplementary spreadsheet File S1). The single-parameter quality metric PQ1 [24] was used to broadly assess the quality of the entire set of structures. PQ1 provides a fractional score reflecting the relative structural quality of a given entry compared to the entire PDB using the following formula:

(1) $$PQ1(\mathrm{entry})=P\left(\frac{P_{R\mathrm{free}}(\mathrm{entry})+P_{\%RSRZ}(\mathrm{entry})+P_{\mathrm{geometry}}(\mathrm{entry})}{3}\right)$$

where $P_{\mathrm{Rfree}},P_{\%\mathrm{RSRZ}}$, and $P_{\mathrm{geometry}}$ are ranking percentiles (the higher the better), characterizing, for a given structural model, its $R_{\mathrm{free}}$, percentage of RSRZ outliers, and the first principal component of the PCA of Ramachandran outliers, Rotamer outliers, and Clashscore, respectively [24]. The percentiles of the entry’s *R*_free_, RSRZ, and the geometry principal component are averaged and ranked against all the entries in the PDB to obtain the final PQ1 score. PQ1 values range from 0 to 1, representing, respectively, the PDB’s lowest and highest quality structures. For this study, PQ1 calculations were performed against all PDB entries determined by X-ray crystallography, available as of 17 March 2025 (192,582 structures).

To assess the quality of small-molecule ligands (other than solvent and ions) interacting with PKA molecules we used the ligand quality score proposed by Shao et al. [46]. The score (currently shown at the RCSB PDB website as Ligand Structure Quality Assessment sliders) assesses a given ligand’s best fit in a PDB structure. The ligand fit is quantified as a combination of the real-space *R* factor (RSR) and the real-space correlation coefficient (RSCC). Similarly to PQ1, the ligand quality score is a fractional value ranging from 0 to 1, representing the lowest and highest quality ligands within the PDB. The ligand quality scores were extracted from the PDB on 17 March 2025, using the RCSB Graph-QL Data API [47].

### 4.3. Comparison of PDB and PDB-REDO Models

A comparison of the PDB and PDB-REDO structures was conducted for a dataset of 289 crystal structures, i.e., all PKA structures available in PDB-REDO. The analyzed metrics included *R*_free_, clashscore, Ramachandran outliers, and rotamer outliers. Since the current version of PDB-REDO (v8.13) does not include clashscore information, we calculated its values using MolProbity [44] as contained in the CCP4 package (version 9.0.004) [45]. For a small subset of structures, we verified that our calculated clashscores are consistent with those reported in the upcoming, but not yet publicly available, PDB-REDO v 8.15. Although official PDB validation reports [46] contain these metrics in the case of PDB structures, we decided to compute them using the same version of MolProbity that we used for PDB-REDO models to achieve full comparability. We noticed some differences between the values we obtained and the ones that were published in the validation reports; however, discussion of this issue is beyond the scope of this paper. For seven structures, the version of MolProbity that we used reported run-time errors and failed to compute the geometrical metrics. The PDB IDs of these structures are listed in Table 1 and more details are given in the comments column of the Supplementary spreadsheet File S1. Comparing the *R*_*work*_/*R*_free_ values between PDB and PDB-REDO was also not straightforward. In addition to the values provided by the depositor, both the PDB and PDB-REDO recalculate *R* and *R*_free_ using validation software from the Data Curation Center (DCC) https://www.dcc.ac.uk/(accessed on 1 April 2025) [48] and Refmac5 [40], respectively. An extensive, interactive comparison of DCC-calculated *R*_*work*_ and *R*_free_ values with the corresponding Refmac5 results from PDB-REDO, is available at: https://bioreproducibility.org/figures/kinase/Figure S1/(accessed on 1 April 2025). Notably, the *R*_*work*_ and *R*_free_ values are generally lower for re-refined structures, highlighting the improvements made by PDB-REDO. However, the values of *R*_*work*_ and *R*_free_ alone are insufficient to fully assess structure quality improvement. The PQ1 score, which could serve as a more comprehensive single-parameter quality estimate, was not calculated due to the absence of RSRZ data in the current version of PDB-REDO. The averages and medians of those metrics are reported in Table 2. We also compared the deposited PDB and re-refined PDB-REDO structures using RMSD values calculated for each pair of models, separately for Cα atoms only, as well as for all non-hydrogen atoms. The RMSD value for the Cα trace was calculated using TM-align (via the tmtools Python package) [49]. Due to the limitations of the original package used for RMSD calculations, we employed PyMOLv. 3161 [50] for the all-atom (non-hydrogen) case and excluded all water molecules from analysis. Performing all-atom RMSD calculations is particularly challenging, as most programs support such alignments only at the level of individual chains rather than complete models. Although PyMOL can perform all-atom alignments, it occasionally fails, and this approach does not always yield reliable results. We identified several problematic cases where PyMOL misaligned the models, producing abnormally high RMSD values—for instance, 3tnp (RMSD = 33.0 Å), or 3l9l and 3l9m (both ≈ 21.5 Å), among others. These outliers should be excluded from any comparative analysis of PDB and PDB-REDO structures.

## Supplementary Material

Supplementary spreadsheet File S1

**Supplementary Materials:** The following supporting information can be downloaded at: https://www.mdpi.com/article/doi/s1, Excel spreadsheet with data discussed in this manuscript

## Figures and Tables

**Table 1.** Classification of the quality of PDB deposits of PKA enzymes.

**Top 10 structures in terms of quality that could not be further improved with PDB-REDO**
5n3l, 5n3o, 6spm, 5n1h, 5n1g, 6spu, 5n3n, 5n3t, 4c36, 5n3q
**Selected structures that were improved by PDB-REDO**
6y89, 7e12, 4dfz, 3ag9, 4yxs, 2qcs, 3kkv, 4ae6, 1q62, 3ovv
**Selected structures for which *R*_free_ increased by 0.02 or more after PDB-REDO**
5n3p, 5n3h, 6sox, 6sq1, 6zn0, 6z44, 5o5m, 6eh0, 5n3r, 5ok3
**Deposits with no structure factors in the PDB, for which fit to the electron density could not be assessed**
2uzu, 2uzw, 2oh0, 2f7e, 2uzt, 2f7x, 2ojf, 2uzv, 2f7z, 1cmk, 1bx6, 1szm, 1stc, 1rek, 1rej, 1jbp, 1jlu, 1xha, 1cdk, 1svh, 1re8, 1xh6, 1sve, 1xh7, 2cpk, 1smh, 1svg, 1xh4, 1xh5, 1xh8, 1veb, 2c1a, 2c1b
***R*_free_ reflections not designated**
1ctp, 3idc, 2qvs, 3o7l, 1ydr, 1yds, 2jdt, 2uvz
**Selected structures exhibiting assorted problems**
3pvb, 3e8c, 3nx8, 3l9m, 3n9l, 3l9n, 4nts
**Structures for which MolProbity (from CCP4 9.0.040) reported run-time errors**
1cdk, 3kkv, 4c36, 4c37, 4c38, 5uzk, 6qj7

**Table 2.** Average and median values of quality metrics of PKA kinase structures deposited in the PDB and re-refined with PDB-REDO.

	*R*_free_	Clashscore	Ramachandran Outliers (%)	Rotamer Outliers (%)
PDB/PDB-REDO–Aver-age	0.2237/0.2143	6.15/3.68	0.32/0.17	3.37/1.51
PDB/PDB-REDO–Me-dian	0.2215/0.2129	3.01/2.62	0.00/0.00	1.41/0.96

## Abbreviations

ADP: Atomic displacement parameter
DCC: Data Curation Center, a Python wrapper for several third-party software programs used for structure analysis
PDB: Protein Data Bank
RMSD: Root-mean-square deviation
RSRZ: Real-space *R*-factor Z-score
